# BindingDB in 2024: a FAIR knowledgebase of protein-small molecule binding data

**DOI:** 10.1093/nar/gkae1075

**Published:** 2024-11-22

**Authors:** Tiqing Liu, Linda Hwang, Stephen K Burley, Carmen I Nitsche, Christopher Southan, W Patrick Walters, Michael K Gilson

**Affiliations:** Skaggs School of Pharmacy and Pharmaceutical Sciences, University of California San Diego, La Jolla, CA 92093, USA; Skaggs School of Pharmacy and Pharmaceutical Sciences, University of California San Diego, La Jolla, CA 92093, USA; Research Collaboratory for Structural Bioinformatics Protein Data Bank, Institute for Quantitative Biomedicine, Rutgers. The State University of New Jersey, Piscataway, NJ 08854, USA; Department of Chemistry and Chemical Biology, Rutgers, The State University of New Jersey, Piscataway, NJ 08854, USA; Rutgers Cancer Institute, Robert Wood Johnson Medical School, New Brunswick, NJ 08903, USA; Research Collaboratory for Structural Bioinformatics Protein Data Bank, San Diego Supercomputer Center, University of California, San Diego, La Jolla, CA 92093, USA; Rutgers Artificial Intelligence and Data Science (RAD) Collaboratory, Rutgers, The State University of New Jersey, Piscataway, NJ 08854, USA; Cambridge Crystallographic Data Centre, Inc., Boston, MA 02108, USA; Deanery of Biomedical Sciences, University of Edinburgh, Edinburgh, EH8 9XD, UK; Relay Therapeutics, Cambridge, MA, 02141, USA; Skaggs School of Pharmacy and Pharmaceutical Sciences, University of California San Diego, La Jolla, CA 92093, USA; Department of Chemistry and Biochemistry, University of California San Diego, La Jolla, CA 92093, USA

## Abstract

BindingDB (bindingdb.org) is a public, web-accessible database of experimentally measured binding affinities between small molecules and proteins, which supports diverse applications including medicinal chemistry, biochemical pathway annotation, training of artificial intelligence models and computational chemistry methods development. This update reports significant growth and enhancements since our last review in 2016. Of note, the database now contains 2.9 million binding measurements spanning 1.3 million compounds and thousands of protein targets. This growth is largely attributable to our unique focus on curating data from US patents, which has yielded a substantial influx of novel binding data. Recent improvements include a remake of the website following responsive web design principles, enhanced search and filtering capabilities, new data download options and webservices and establishment of a long-term data archive replicated across dispersed sites. We also discuss BindingDB’s positioning relative to related resources, its open data sharing policies, insights gleaned from the dataset and plans for future growth and development.

## Graphical Abstract

**Graphical Abstract:**
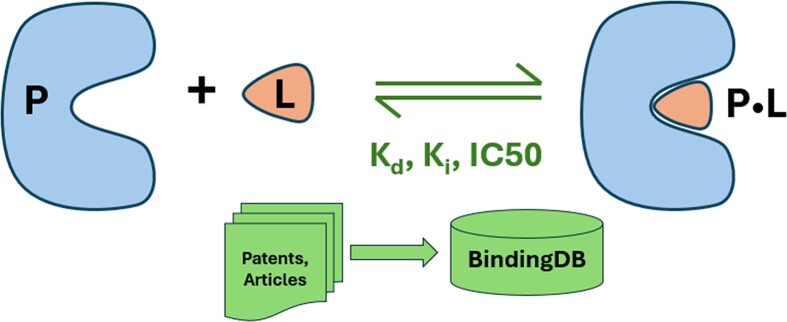

## Introduction

Most new FDA-approved medications are small, organic molecules (1) and the identification of a small molecule, or ligand, that binds a targeted protein with sufficient affinity is an early step in many drug discovery projects. This process involves considerable trial and error, and a typical drug discovery campaign project requires measuring the affinities of hundreds or thousands of compounds. Although the resulting binding data are essentially a by-product of an effort aimed at generating just a few potent binders, some of which will be advanced through the discovery pipeline, they are still extremely useful. First, data on the compounds that bind a given protein can inform the discovery of ligands for similar proteins. More interestingly, the aggregation of data from drug discovery projects spanning many protein targets has *emergent* value for a range of applications, including, for example:

- Developing new insights into the principles of medicinal chemistry and drug design.
- Testing and training physics-based computer-aided drug discovery (CADD) methods and artificial intelligence (AI) methods.
- Annotating proteins in signaling and metabolic pathways with known binders as possible chemical probes and drugs.
- Predicting the protein targets of bioactive small molecules, based on chemical similarity.

In 1997, a workshop involving the National Institute of Standards and Technology (NIST) and Rutgers University was held to plan the development of a publicly accessible database of measured non-covalent binding data. Initial funding by NIST and the National Science Foundation (NSF) supported the first steps of this effort, and a 2004 grant from the National Institutes of Health (NIH) enabled initiation of BindingDB (bindingdb.org), the first public, web-accessible, database of quantitative protein-ligand affinity data (2). Currently, funded by the NIH/NIGMS and based at University of California San Diego, BindingDB provides open access to a growing dataset comprising 2.9 million experimental protein-small molecule binding data points, which span 1.3 million compounds and thousands of protein targets.

Building on prior reports in this journal from 2007 (3) and 2016 (4), we now provide a 2024 update of BindingDB, addressing the structure of the dataset and its continuing expansion, its positioning relative to related resources, its interface capabilities and usage patterns and insights into the dataset itself.

## The BindingDB dataset

### Binding affinities are organized into one entry per document

**Figure 1.**
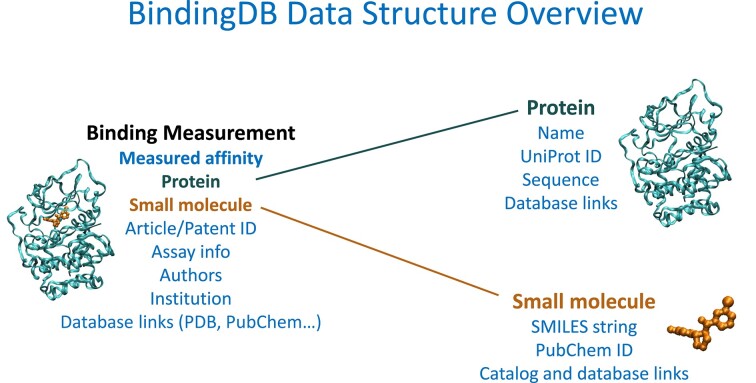
Diagram of the core data structure of BindingDB. A typical binding measurement is a measured affinity between a protein and a small molecule and was extracted from an article or a patent which documents the assay method and is associated with authors working at a given institution. The measurement may correspond to data in e.g. the PDB in the form of a cocrystal structure. Details of the protein target include a name and synonyms, a UniProt ID and its sequence, while the small molecule ligand is defined by a SMILES string and, typically a PubChem SID. Many compounds are also associated with links to chemical catalogs and entries in other small molecule databases. BindingDB also contains smaller collections of protein-protein and host-guest binding data.

The vast majority of the data in BindingDB are experimentally measured affinities of well-defined proteins, termed targets, with small organic molecules, termed compounds or ligands. However, BindingDB also contains small collections of protein-protein and host-guest binding affinities, as well as isothermal calorimetric and kinetic binding data. The data are organized into entries, where each entry is typically associated with a single scientific article or a single pending or granted patent. Thus, the data in one entry generally come from a single institution and are measured in the same manner. Each entry is assigned its own DOI with a defined landing page; for example, the BindingDB DOI 10.7270/Q2H993SB resolves to https://www.bindingdb.org/entry/5649.) Binding affinities are typically expressed in terms of concentrations, most frequently as an IC50, an EC50, a dissociation constant (Kd), or an inhibition constant (Ki) expressed in nanomoles/L (nM). In all cases, a lower value implies a higher affinity. A given binding measurement pertains to a given protein target, a given compound, a description of the assay used, and the authors, institution (e.g. a university or company), publication information (e.g. a PubMed ID or a US patent number) and any other information associated with the entry (Figure 1). BindingDB curation procedures also add annotations of entries with additional information, such as the identities of co-crystal structures available in the Protein Data Bank (5–9), the UniProt ID of the target (10–12) and compound links to chemical database and commercial chemical catalogs. BindingDB does not curate experimental three-dimensional structure data, instead providing users with links to the RCSB PDB (9) for available structures of protein–ligand complexes, based on the chemical identity of the ligand and the sequence identity of the protein.

### The BindingDB dataset is growing rapidly

**Figure 2.**
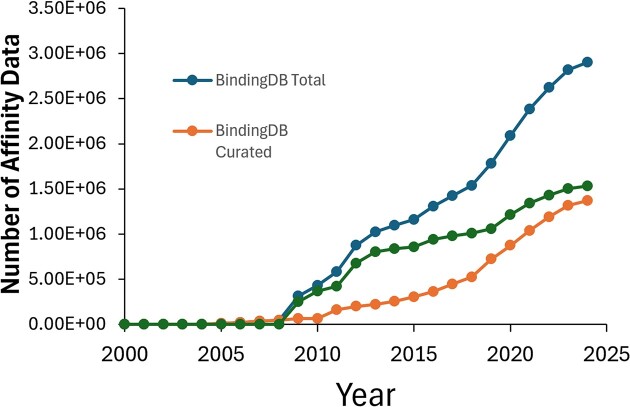
Number of affinity measurements in BindingDB 2000–2024. The dataset comprises data curated by BindingDB and data imported from ChEMBL, as detailed in the text. The leveling in 2024 primarily reflects the fact that the year is not yet over.

The growth of the BindingDB dataset since its inception is illustrated in Figure 2. Since our last published report (4), the number of data in BindingDB that were curated by our team has grown more than fourfold, and the total number of data in BindingDB grew more than three-fold. Approximately half of the measurements presently in BindingDB were extracted from US patents and scientific articles by BindingDB staff, and most of the remaining data in BindingDB were imported from ChEMBL (13–15), which has a robust curation effort focused on the scientific literature, as discussed below. The most recent 2-year's flow of data into BindingDB is analyzed in Table 1, which shows that BindingDB curators added about 250 000 new protein-ligand binding data during this period, mainly from US patents. Importation of suitable data from ChEMBL added another 101 000 data, mainly from articles. Each new tranche of data curated by BindingDB is released monthly, with updated data files available for download around the start of each calendar month. Suitable data from each new ChEMBL update (e.g. ChEMBL 34) are identified, annotated and imported typically within a month of their initial release.

**Table 1.** Breakdown of the main data flows into BindingDB over the last 2 years (7/1/2022- 6/30/2024), in terms of numbers of curated documents (articles or patents), protein targets, compounds, new compounds (i.e. compounds not previously present in BindingDB) and affinity data^1^

Source	Documents	Targets	Compounds	New compounds	Affinity data
**BDB US Patents**	1443	1000	146 681	117 377	241 430
**BDB WIPO Patents**	42	47	2394	2394	3017
**BDB Other**	12	11	4335	2893	5003
**BDB Total**	**1497**	**1016**	**153 374**	**122 059**	**249 450**
**ChEMBL 32 Articles**	491	703	9044	7966	20 489
**ChEMBL 33 Articles**	1383	1369	24 662	20 183	53 019
**ChEMBL 34 Articles**	770	1057	14 546	10 522	27 662
**ChEMBL Total**	**2644**	**3129**	**48 252**	**38 671**	**101 170**
**Total**	**4141**	**4145**	**201 626**	**160 730**	**350 620**

^1^Rows marked BDB list data curated by the BindingDB project. Rows ChEMBL 32, 33 and 34 list the numbers added by each successive ChEMBL release. Totals are provided for BindingDB curation, all ChEMBL importation and across both projects.

### The BindingDB dataset is related to other widely used databases

It is useful to place BindingDB into the context of related database efforts. The ChEMBL project has similar aims but a different ambit, as it collects not only data with an assigned, single protein target, but also phenotypic assay results and ADMET data. In addition, ChEMBL focuses on curation of data from scientific articles, while BindingDB focuses on curation from patents. As noted in Section 2.2, BindingDB imports only those data from ChEMBL that fit the BindingDB curation criterion of having a well-defined protein target, while ChEMBL has imported a subset of the data curated by BindingDB, as detailed below. As of this writing, BindingDB has imported all suitable ChEMBL data up to ChEMBL 34, and ChEMBL includes the data from about 1900 US patents curated by BindingDB.

**Figure 3.**
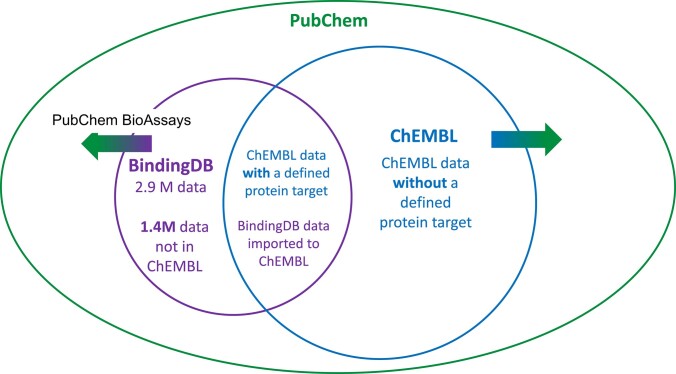
Relationships among data collections of BindingDB, PubChem and ChEMBL. BindingDB and ChEMBL both maintain robust data curation efforts. BindingDB focuses on measured protein-small molecule affinities, while ChEMBL also includes a wide range of small molecule activities measured in phenotypic/biological assays. BindingDB and ChEMBL data are provided to PubChem in the form of PubChem BioAssays. PubChem includes an even wider array of small molecule data types but does not extract data from documents as done by BindingDB and ChEMBL. BindingDB and ChEMBL also make their data available to each other for redistribution. From ChEMBL, BindingDB imports only those data with a well-defined protein target, as opposed to biological activity data where the target is not defined.

The PubChem database (16–20), which is operated by the US National Institute of Health's National Library of Medicine (NLM), is a global hub integrating bioactivity and chemistry data. It currently contains 118 million compound structures from over 1000 sources (pubchem.ncbi.nlm.nih.gov/docs/statistics). PubChem operates a submission model with no internal curation but housing bioactivity data for over 4 million compounds from many curated resources, including ChEMBL and BindingDB as two of the largest contributors Figure 3. By collaborative arrangement, the PubChem team downloads a MySQL data dump from BindingDB on a monthly basis and integrates these data into PubChem. Those compounds without an existing PubChem substance ID (SID) are assigned one and compounds new to PubChem are assigned compound IDs (CIDs) (pubchem.ncbi.nlm.nih.gov/docs/compound-vs-substance), while the assay data are used to create PubChem BioAssays, which are identified by Assay IDs. Thus, if a BindingDB entry contains only one assay type, then all the data in the entry go into a single PubChem BioAssay. However, if an entry contains data from multiple different assays, such as for inhibition of various enzymes, then the data from each assay within the entry are mapped to its own separate PubChem BioAssay. BindingDB subsequently downloads and integrates the new SIDs, CIDs and AIDs. As of 5 August 2024, BindingDB has supplied 13 514 BioAssays, and 1 269 104 distinct compounds (represented by CIDs). Because each BindingDB entry is associated with an article DOI, users viewing an article on the NLM’s PubMed site can choose the ‘LinkOut- more resources’ link in the right-hand margin to navigate directly to the corresponding data in BindingDB.

Two closely related global efforts, SureChEMBL and PDBBind, also deserve mention. SureChEMBL (21) is a project that collects compounds from patents and makes them searchable and downloadable. Its automated curation platform enables extensive coverage of existing patents but does not associate compounds with binding affinity data and therefore does not overlap with BindingDB. PatCID (22) is a recently announced resource that is similar in spirit to SureChEMBL. The PDBBind database (23–25) collects protein-small molecule crystal structures from the Protein Data Bank and joins them with their respective binding affinity data in order to provide a curated dataset uniting three-dimensional structures and affinities for ∼23 000 protein–ligand complexes (https://www.pdbbind-plus.org.cn/, accessed 7/3/2024). This number is much smaller than the 2.9 million measurement scope of BindingDB because cocrystal structures have been determined for only a fraction of protein-ligand pairs for which affinity data are available. In particular, whereas BindingDB contains binding data for many congeneric ligand series, PDBBind is expected to contain data for only one or a few compounds from any given congeneric series. On the other hand, PDBBind contains binding data for a number of protein-ligand complexes in the PDB that are not present in BindingDB, making these complementary resources. It is also worth mentioning PLINDER, a new collection of protein-small molecule structural data (but not affinities) designed for training and testing AI methods (26).

BindingDB also integrates with the UniProtKB protein knowledgebase (10,12,27) and the RCSB Protein Data Bank (PDB) (7,9). BindingDB assigns UniProtKB IDs to protein targets and uses UniProtKB’s recommended protein names. UniProtKB in turn provides links from their protein records to corresponding targets in BindingDB, with their associated small molecule binding data. We also scan each protein–ligand pair in BindingDB against the protein-ligand structural data in the RCSB PDB looking for exact compound matches, and identify two sets of relevant structures. In one set (15 328 structures), the protein sequence identity is above 85%, and in the other (10 126 structures), it equals 100%. (See www.bindingdb.org/rwd/bind/ByPDBids.jsp and www.bindingdb.org/rwd/bind/ByPDBids_100.jsp.) Links to these structures are provided in various locations on the BindingDB website and in search results. The RCSB PDB, in turn, uploads a record of the >85% match set and provides links from these structure entries to the corresponding binding data in BindingDB.

### BindingDB curates a large flow of data from US patents

Currently, BindingDB curation activities provide the largest single flow of protein-ligand binding data into public domain databases. We initially focused on extracting data from scientific articles, but the establishment of ChEMBL’s excellent curation of key journals led to a risk of overlapping effort. In addition, different articles across different journals present their data in different formats, making it harder to develop a single, seamless, curation pipeline. Therefore, approximately a decade ago, we began to explore the curation of patents, which had been largely untapped by public curation efforts. It became clear that US patents contain substantial relevant data and follow a more standardized format than articles and are amenable to greater automation. Today, BindingDB’s curation of data from US patents is distinctive within the ecosystem of open-source knowledgebases, unlocking a continuous flow of FAIR protein-ligand binding data that has minimal overlap with that provided by other curation efforts. Over the last 2 years, this effort has processed about 125 000 affinity data per year (Table 1).

Each week, BindingDB downloads the US patent ‘redbook’, a data set of newly published patent applications and newly granted patents (https://bulkdata.uspto.gov/data/patent/grant/redbook/2024/, https://bulkdata.uspto.gov/data/patent/application/redbook/2023/) in XML format. We then use purpose-built software to identify patents likely to contain suitable data. Criteria include categorization into the Cooperative Patent Classification (CPC) subclasses that have a high specificity for medicinal chemistry SAR filings, notably C07 (Organic Chemistry) and A61 (Preparations for Medical, Dental or Toiletry Purposes), presence of so-called complex work units with molecular structures and presence of tabular data associated with key words such as ‘IC50’ and ‘Kd’.

**Figure 4.**
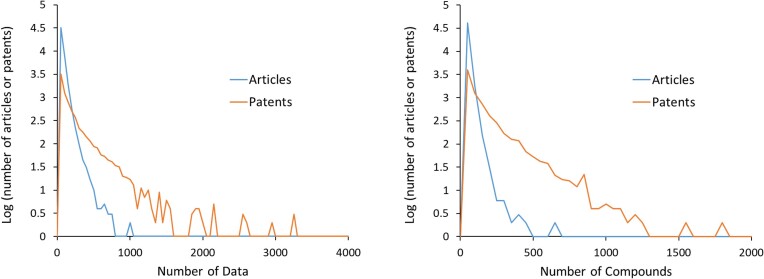
Histograms of the numbers of binding data (left) and compounds (right) curated from articles and patents, for all non-categorical data (e.g. ‘IC50 < 1 μM’) in BindingDB. The histogram bins are of width 50 and bin counts are plotted as log10(N) for N > 0 and 0 for N = 0.

These patents are automatically loaded into HTML-based forms which display a preliminary curation, including association of compounds with tabular data. A trained curator compares the preliminary curation with the patent document, correcting data for curatable patents as needed, and rejecting patents that turn out not to have suitable data. (For example, as of mid-2024, patents containing only binned affinity data, such as ‘IC50 < 1 μM’, will be rejected.) Curated data from this step are loaded into a staging server, where they are compared against the patent documents by a second curator and any concerns are resolved in consultation with the first curator. BindingDB curation software is then used to annotate the curated data by, for example, identifying appropriate links to the PDB and UniProt, and the data are then migrated to the public database server. The compounds are submitted to PubChem and, a week later the resulting PubChem SIDs and CIDs are obtained and associated with the BindingDB data. This largely automated process allows a small staff to curate substantially all suitable newly granted US patents and new pending patent applications in each weekly download. This level of efficiency is essential, because the average number of data per patent in BindingDB is 160, while the average number per article is 40. This difference is reflected in Table 1, where BindingDB curation of about 1500 patents yielded about twice as many affinity data as ChEMBL curation of about 2600 articles. Figure 4 provides a more detailed look at the distributions of binding data per article and per patent (left) and of compounds curated per article and per patent (right), across all of BindingDB’s holdings. Articles with very high data counts may include, for example, very large reports of new structure–activity relationships (SAR) for a given protein target (28); reports of new data for many compounds against a panel of kinases (29); large ADME-related studies (30,31); publications associated with datasets within the PDSP database (32,33); and review articles or articles with large datasets assembled for quantitative structure-activity studies (34).

Although we monitor the applicability of rapidly emerging AI technologies to curation, we are not aware of any current AI approach that would remove the need for human curation, and US patents are already so well structured that preprocessing by AI would add little if any value. As of this writing, we are curating downloads from mid-2024 and working forward.

Although our curators primarily target US granted patents and US pending patents, some scientific articles of particular interest are curated. For example, in response to the COVID-19 pandemic, we collected data for SARS-CoV-2 and other coronaviruses from articles. These data are highlighted in a dedicated web page, https://www.bindingdb.org/rwd/bind/Covid19.jsp. We also curate selected recent WIPO (https://www.bindingdb.org/rwd/bind/ByWIPO.jsp) patents of particular interest, such as for SARS-Cov-2, because data often appear in WIPO patents several years before they appear in US patents. However, we focus on US patents because their formatting is more amenable to semi-automated curation.

### Curation errors appear to be uncommon

Any errors present in the BindingDB dataset may be grouped into two broad categories. The first category, errors in the source document (typically a patent or scientific article), may result from experimental errors of various types, errors in the analysis of raw data, or clerical errors by authors and editors. Such errors are difficult for us to discover and hence correct, though they might be flagged as outliers in cases where the same protein-ligand affinity has been measured more than once. Because different measurement procedures can introduce different biases, it is likely that the measured *relative* affinities of a series of ligands for a given protein – i.e. ratios of K_d_s or IC50s – as measured by one method in one lab are more accurate than the affinities themselves. The second category results from clerical errors in curation or data management, mainly by BindingDB or ChEMBL. These appear most commonly as a factor of 1000 error resulting from miscuration of e.g. an IC50 value as nanomolar instead of micromolar or *vice versa*. Based on user feedback, the next most common error appears to be misidentification of a protein target. This usually occurs during curation of a patent or article with data on multiple targets and/or subtypes of a target.

BindingDB web pages allow users to report apparent errors, which we check and correct when needed. Encouragingly, we receive fewer than 10 error reports per year. In addition, for a number of years, when new data from scientific articles appeared in BindingDB, we invited the corresponding authors of the articles to check our version of their data for errors. However, we received virtually no responses, so we ended this practice.

### Scientific characteristics and features of the dataset

Out of the 2.9 million binding data currently in BindingDB, the vast bulk, i.e. 2.4 million, are associated with human-derived protein targets. The next most represented source organisms are rat (177 K data), mouse (65 K) and human immunodeficiency virus type 1 (29 K), followed by a long tail of ∼560 additional source organisms with fewer and fewer binding data. Human-derived protein targets number 3410. Table 2 lists the 25 human-derived targets with the most data in BindingDB. These comprise primarily signaling kinases, cell-surface receptors, nuclear hormone receptors and other enzymes.

**Table 2.** The 25 human-derived protein targets with the most binding data in BindingDB, showing the number of measured affinities for each target^1^

Target	UniProt ID	Number of affinities
Proto-oncogene tyrosine-protein kinase receptor Ret	P07949	26810
Tyrosine-protein kinase JAK2	O60674	26262
Epidermal growth factor receptor	P00533	24745
Tyrosine-protein kinase BTK	Q06187	23244
Bromodomain-containing protein 4	O60885	21446
Tyrosine-protein kinase JAK1	P23458	21217
Nuclear receptor ROR-gamma	P51449	18532
Potassium voltage-gated channel subfamily H member 2	Q12809	17918
Phosphatidylinositol 4,5-bisphosphate 3-kinase catalytic subunit delta isoform	O00329	17663
Non-receptor tyrosine-protein kinase TYK2	P29597	17097
D(2) dopamine receptor	P14416	16573
Beta-secretase 1	P56817	16016
Tyrosine-protein kinase JAK3	P52333	14947
Carbonic anhydrase 2	P00918	14542
Vascular endothelial growth factor receptor 2	P35968	14231
Phosphatidylinositol 4,5-bisphosphate 3-kinase catalytic subunit alpha isoform	P42336	13828
Histone deacetylase 1	Q13547	13751
Isocitrate dehydrogenase [NADP] cytoplasmic	O75874	13499
Orexin receptor type 2	O43614	13360
Cyclin-dependent kinase 2	P24941	13328
Interleukin-1 receptor-associated kinase 4	Q9NWZ3	13303
Sodium channel protein type 9 subunit alpha	Q15858	13133
GTPase KRas	P01116	13088
Cannabinoid receptor 2	P34972	12491
Carbonic anhydrase 1	P00915	12457

^1^Note that a given target row may implicitly include multiple mutant forms of the protein, such as P07949[G810S], P07949[M918T] or P07949[1-999,M918T].

**Figure 5.**
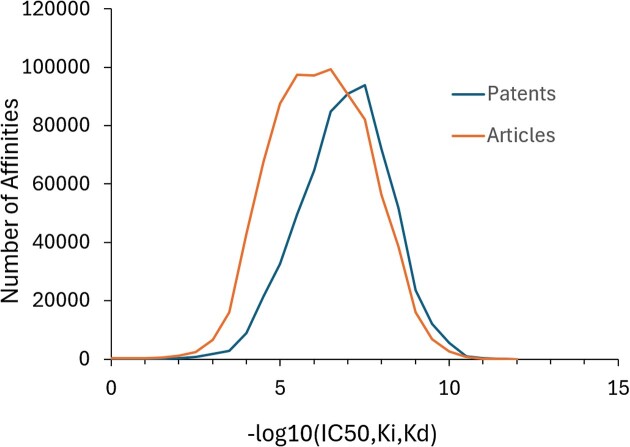
Distributions of affinities from patents and articles. Data of types IC50, Ki and Kd (M) are combined. Binned or categorical data (e.g. ‘IC50 < 1 μM’) are omitted.

**Figure 6.**
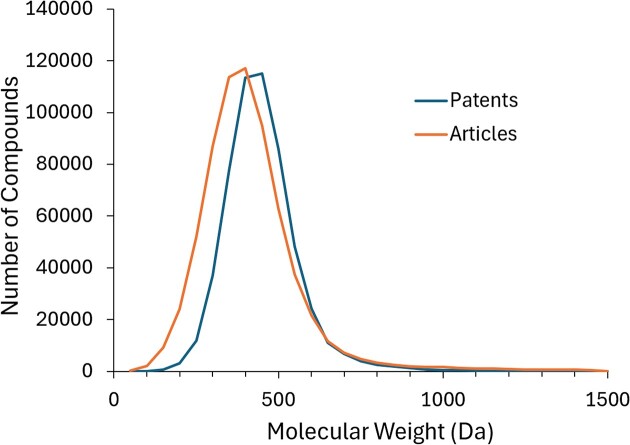
Histograms of molecular weights of compounds in BindingDB extracted from patents and articles.

Most affinity data stored in BindingDB are reported and hence curated as IC50s (1.8 million), followed by K_i_ values (560 K), EC50s (220 K) and K_d_ values (100 K). Interestingly, affinities curated from patents tend to be greater (stronger binding) than those from articles, as shown in Figure 5, with a ∼10× lower mode in the distribution of IC50, K_i_ or K_d_ values. In addition, molecular weights of compounds extracted from patents tend to be higher than those of compounds coming from articles (Figure 6), with a mode of ∼450 versus ∼400 Da. These differences presumably reflect the fact that patents present more chemically elaborated ligands than those described in articles, which are more exploratory in character.

**Figure 7.**
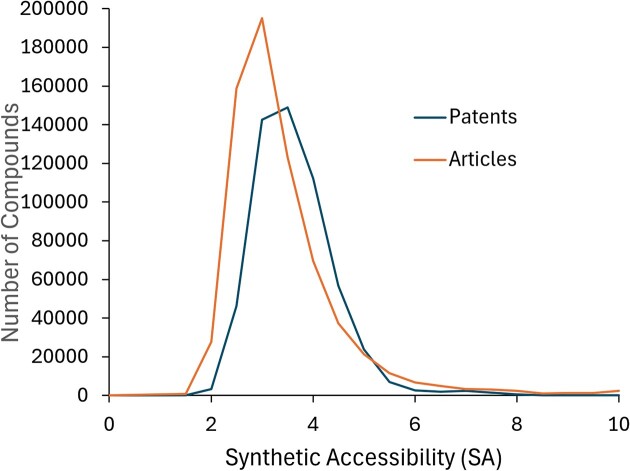
Histograms of estimated SA of BindingDB compounds extracted from patents and articles, computed with RDKit (see text).

Applications of generative AI methods that propose drug-like ligands (35–45) frequently assess the quality of their generated ligands in terms of the quantitative estimate of drug-likeness (QED) (46), on a scale of 0–1. The distribution of QED values for the compounds in BindingDB peaks at about 0.5, for compounds drawn from both patents and articles. This result is similar to that initially reported for an early version of ChEMBL (46). Generative AI methods also frequently use measures of synthetic accessibility (SA) to guide the generation of compounds that are amenable to chemical synthesis. As shown in Figure 7, a metric of SA available in the 3/2023 release of RDKit (RDKit: Open-source cheminformatics, https://www.rdkit.org; (47)) peaks at about 3, on a 0–10 scale, for all compounds in BindingDB, indicating non-trivial synthetic challenges for most compounds. Interestingly, compounds in patents are scored as slightly more synthetically accessible, on average, than compounds in articles.

**Figure 8.**
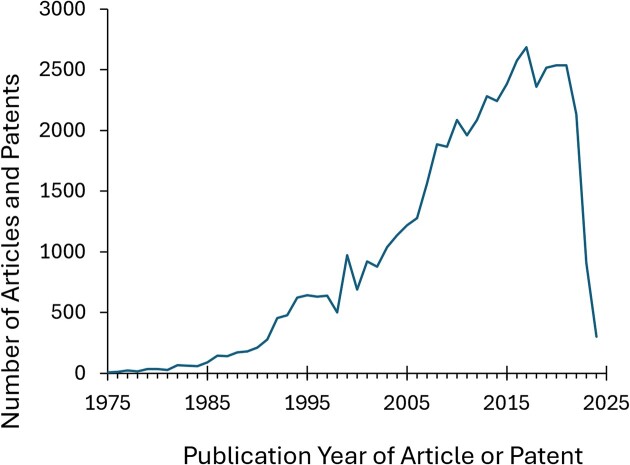
Histogram of publication dates of the articles and patents curated into BindingDB from all sources.

Most of the data in BindingDB derive from documents (articles and patents) published within the last 10 years or so, as show in Figure 8. This reflects the efforts of both BindingDB and ChEMBL to curate relatively current documents, as well, presumably, as the rising number of data published per year.

## BindingDB provides tools for diverse users

The most recent survey of BindingDB users, which occurred in 2020, indicated that about 80% work in academia, with the rest spread across government, industry and other/no affiliation. When allowed to select multiple application areas, 75% said they use BindingDB for drug discovery, 30% for chemical biology, 25% for development of physics-based models, 15% for machine learning/AI training and testing, 15% for systems biology and 10% for toxicology. Again, allowing multiple responses, 75% said they browse/query the website, 45% download data, 15% integrate the downloaded data with a local database and 10% use BindingDB webservices.

**Figure 9.**
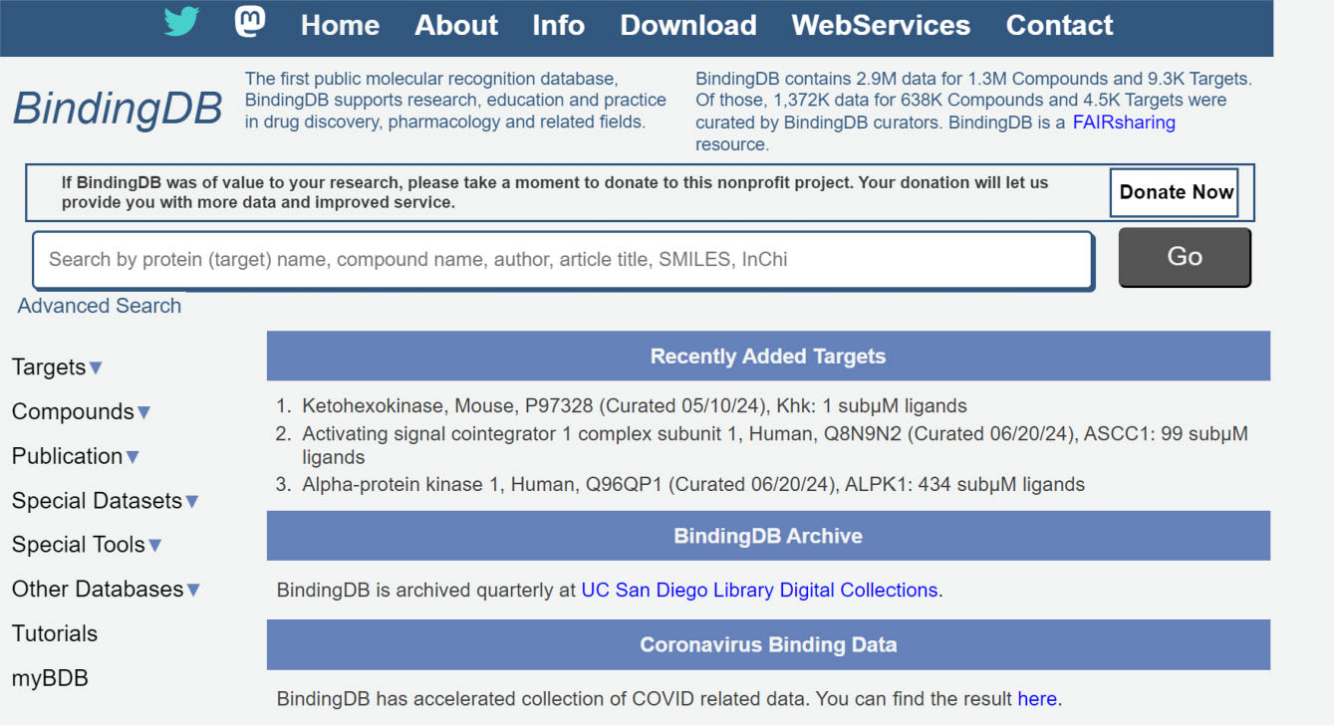
BindingDB homepage, shown the left-hand menu (targets, compounds, publications, etc.).

**Figure 10.**
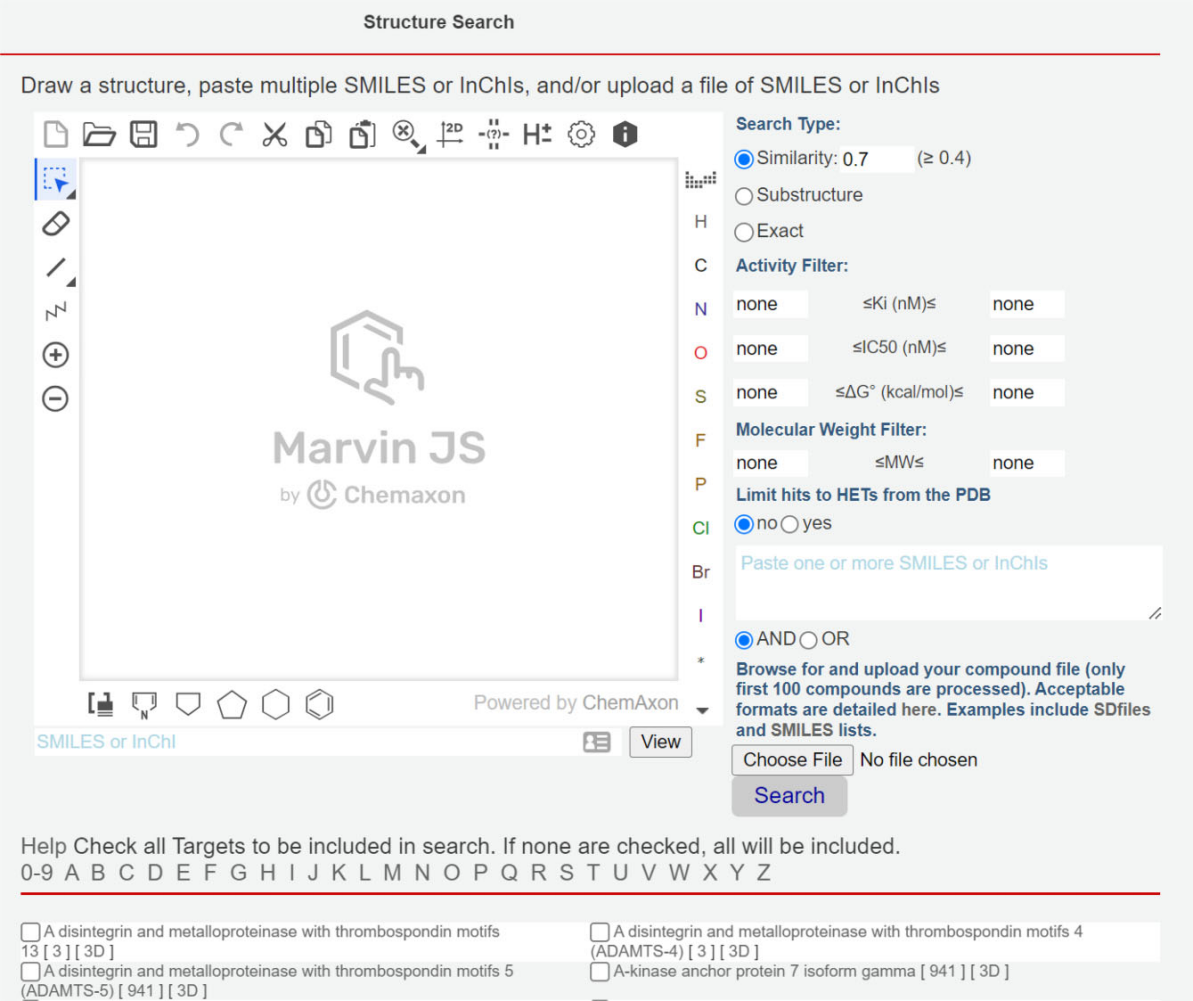
Chemically aware searching in BindingDB enabled by ChemAxon tools. Note that one can search for exact compound matches, for compounds a given chemical substructure or similar compounds. One may also refine the search based on affinity limits, molecular weight or presence of the compound in the PDB, and one may limit the search to one or more targets by using the checkboxes below the chemical draw window.

BindingDB provides a range of tools to support these and other applications. Many are accessed *via* the left-hand menu available on most of our webpages (Figure 9). For example, one can readily access all data available for a given protein target by browsing target names (https://www.bindingdb.org/rwd/bind/ByTargetNames.jsp) or UniProt IDs (https://www.bindingdb.org/rwd/bind/ByUniProtids.jsp). For each target, a tab-separated value (TSV) file with SMILES (48) and InChI (49) strings as well as SDfiles with 2D or computed 3D structures, is available for immediate download. Alternatively, one may view the data online in a tabular format, which has been described previously (4). One can view or download a prepared file of all data from a given article (https://www.bindingdb.org/rwd/bind/ByJournal.jsp, https://www.bindingdb.org/rwd/bind/ByPubMed.jsp) or patent (https://www.bindingdb.org/rwd/bind/ByPatent.jsp, https://www.bindingdb.org/rwd/bind/ByWIPO.jsp). Integrated ChemAxon tools allow chemically aware searching within BindingDB, optionally in conjunction with filtering by affinity range, existence of the compound in the PDB and/or in relation to any selection of protein targets (Figure 10).

The download page (https://www.bindingdb.org/rwd/bind/chemsearch/marvin/Download.jsp) provides date-stamped prepared downloads that include database dumps, TSV files and SDfiles, for the whole database and data subsets, such as all data curated by BindingDB. In addition, we provide RESTful webservices that return data in either XML or JSON format (https://www.bindingdb.org/rwd/bind/BindingDBRESTfulAPI.jsp).

Further details of the overall structure and capabilities of the BindingDB website are available in our previous update (4). The following subsection highlights more recent enhancements.

### BindingDB has implemented new features and capabilities

**Figure 11.**
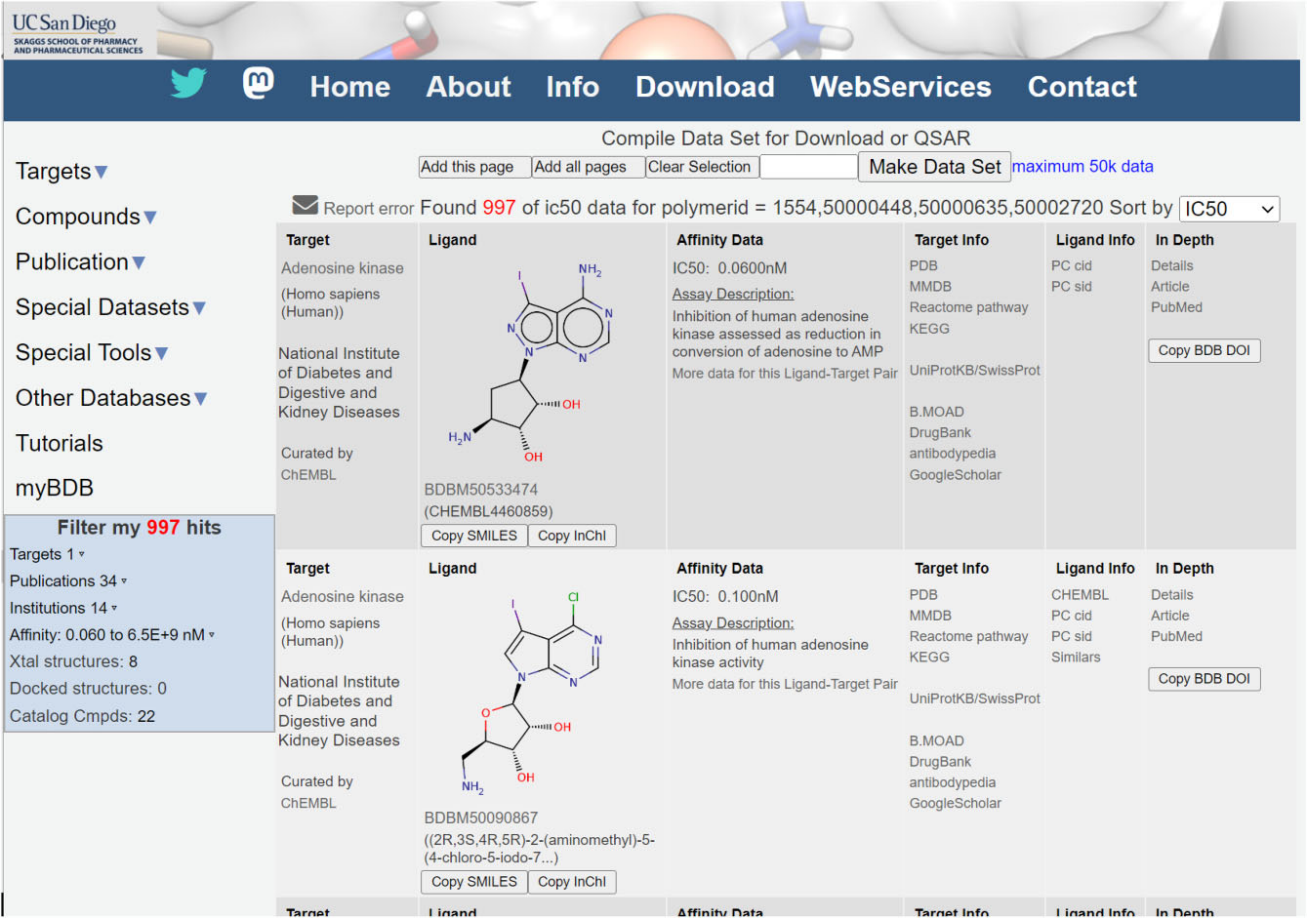
Sample BindingDB search result, showing the new filter options in the left margin.

Leading features and capabilities added since our 2016 update in this journal (4) include the following: Figure 11

- **Responsive Web Design**: The formats of the main page types now use responsive web design (50) to adapt gracefully to different screen widths, from desktop monitors to cell phones. The new page design also simplifies retrieval of SMILES and InChI strings.
- **Results Filters**: BindingDB’s tabular presentation of search results (Figure 11) has been outfitted with a filter tool, which allows users to slice out search results along various dimensions. For example, the filter tool in Figure 11 shows that all 997 data pertain to a single protein target but derive from 34 publications; clicking on ‘34’ yields a pull-down menu of the publications which allows viewing the data from each individual publication. Similarly, one may select the data according to the 14 institutions where the various publications originated; select data within a given affinity range; choose the eight protein–ligand pairs with a corresponding co-crystal structure in the Protein Data Bank; or choose the 22 results for which BindingDB has identified that the ligand is commercially purchasable. These filtration options are particularly useful to refine an initial search or browse operation that has generated an excessively large set of results.
- **Improved Browsing by Target Name**: The list of protein targets by name (bindingdb.org/rwd/bind/ByTargetNames.jsp) has been enhanced by providing a header entry for each name followed by a separate row for each source organism and mutant. This feature is designed to help users locate data of interest more precisely and quickly.
- **PubChem BioAssay Submissions:** We initiated the complete and regular upload of all BindingDB data to PubChem in the form of Bioassays. This procedure has so far generated 13 541 Assay IDs, of which 10 210 come from patents.
- **Enhanced and Expanded Data Downloads**: The prepared data downloads (www.bindingdb.org/rwd/bind/chemsearch/marvin/Download.jsp) now include both Oracle and mySQL data dumps, and all files are now annotated with dates and md5 checksums. As noted above, these files are normally updated near the start of each calendar month.
- **Archiving of CSAR and D3R Datasets**: BindingDB provides stable access to the protein-ligand datasets developed and used in the course of the CSAR (51,52) and D3R (53–56) prediction challenges (www.bindingdb.org/rwd/bind/ByD3R.jsp).
- **Webservice to Obtain Data by PDB Entry ID**: A new webservice supports retrieval of BindingDB data related to specific protein structures in the PDB (www.bindingdb.org/rwd/bind/BindingDBRESTfulAPI.jsp). This provides all binding data that meet user-defined affinity and protein sequence identity criteria. That is, the protein target must have a sequence identity with the PDB protein above a user-specified threshold, and the binding affinity of the compound must be greater than a second user-specified threshold…
- **Long-term Archiving of BindingDB Data**: A long-term, open-access, archive of BindingDB data has been established within the UC San Diego Library Research Data Collections. The contents of the Digital Collections in turn are preserved in Chronopolis (57), a dark preservation system that includes node partners in Texas and Maryland. BindingDB cuts a new archival version, which includes detailed metadata, on a quarterly basis (https://doi.org/10.6075/J0HD7VVF).
- **Creation of a Browser Extension Linking Articles and Patents to Data**: We developed a novel web browser extension which, when installed in Chrome, Firefox, Edge or Brave, flags the user when they are viewing an article or patent that corresponds to an entry in BindingDB and provides links to view the data at bindingdb.org and/or download the data in the form of a TSV file containing one compound SMILES string per row, along with the corresponding target and binding data. This advance is made possible by the fact that the metadata of articles and patents online include DOIs and patent numbers, and these map to BindingDB entries.
- **A Python Script for the Analysis of Structure-Activity Relationships in BindingDB Data**: We (PW) developed an interactive tutorial illustrating the use of open-source software tools to extract SAR from patent data in BindingDB. This employs a Jupyter notebook to guide users through the process of downloading and analyzing patent data. It can be run on the popular Google Colab platform, eliminating the need for local software installation. The tutorial highlights several valuable capabilities, such as examining activity distributions, utilizing Bemis–Murcko scaffolds (58) to comprehend patent scope, and targeting key substituents through R-group decompositions. See https://colab.research.google.com/github/PatWalters/practical_cheminformatics_tutorials/blob/main/patent/patent_analysis.ipynb.

## BindingDB is openly shared and well documented

BindingDB is listed as a FAIRsharing resource (https://doi.org/10.25504/FAIRsharing.3b36hk), and data curated by BindingDB are shared under the Creative Commons Attribution 4.0 License (CC BY 4.0). This allows both non-commercial and commercial reuse and redistribution, subject only to citation of BindingDB and notation of any changes made to the data. Data curated by ChEMBL, which is also a FAIRsharing resource, are shared under the Creative Commons Attribution-ShareAlike 3.0 license (https://chembl.gitbook.io/chembl-interface-documentation/about, accessed 7/3/2024). This adds the requirement that any reused ChEMBL data must be redistributed under the same license. Thus, ChEMBL data in BindingDB are subject to the ChEMBL license, and they are marked as curated by ChEMBL to enable compliance. In contrast, the user license for PDBBind forbids redistribution without explicit permission as of this writing (http://www.pdbbind.org.cn/enroll.php), and we do not collect the PDBBind dataset. The BindingDB webpages provide documentation of the formats of our downloads (e.g. TSV files and SDfiles), and of the webservices provided.

Our open-access policy enables widespread use of BindingDB. During the year prior to this writing, users downloaded the Oracle and mySQL data dumps 1400 and 1000 times, respectively, and there were also over 300,000 downloads of other comprehensive data files. The use of emerging AI methods for drug discovery has led to strong interest in the BindingDB dataset for both training and testing of AI methods, contributing to the approximately 400 citations per year of our prior articles about BindingDB (2–4).

## Directions

In the coming years, BindingDB will continue to curate measured protein-ligand binding data, generating a unique flow of machine-readable, freely licensed, information for use by the worldwide research community. We also plan to improve the reliability and response times of the BindingDB website via hardware and software upgrades, as well as improved software documentation, with the ultimate goal of becoming a CoreTrustSeal (59,60) certified resource.

## Data Availability

Data curated by BindingDB are shared under the Creative Commons Attribution 4.0 License (https://creativecommons.org/licenses/by/4.0). Data in BindingDB that were curated by ChEMBL are shared under the Creative Commons Attribution-ShareAlike 3.0 Unported license (http://creativecommons.org/licenses/by-sa/3.0).

## References

[1] Mullard A. 2020 FDA drug approvals. Nat. Rev. Drug Discov. 2021; 20:85–90. 33402709 10.1038/d41573-021-00002-0

[2] Chen X. , Liu M., Gilson M.K. BindingDB: a web-accessible molecular recognition database. Comb. Chem. High Throughput Screen. 2001; 4:719–725. 11812264 10.2174/1386207013330670

[3] Liu T. , Lin Y., Wen X., Jorissen R.N., Gilson M.K. BindingDB: a web-accessible database of experimentally determined protein–ligand binding affinities. Nucleic Acids Res. 2007; 35:D198–D201. 17145705 10.1093/nar/gkl999 PMC1751547

[4] Gilson M.K. , Liu T., Baitaluk M., Nicola G., Hwang L., Chong J. BindingDB in 2015: a public database for medicinal chemistry, computational chemistry and systems pharmacology. Nucleic Acids Res. 2016; 44:D1045–D1053. 26481362 10.1093/nar/gkv1072 PMC4702793

[5] Bernstein F.C. , Koetzle T.F., Williams G.J., Meyer E.F. Jr., Brice M.D., Rodgers J.R., Kennard O., Shimanouchi T., Tasumi M The Protein Data Bank: a computer-based archival file for macromolecular structures. J. Mol. Biol. 1977; 112:535–542. 875032 10.1016/s0022-2836(77)80200-3

[6] Berman H.M. , Westbrook J., Feng Z., Gilliland G., Bhat T.N., Weissig H., Shindyalov I.N., Bourne P.E. The Protein Data Bank. Nucl Acids Res. 2000; 28:235–242. 10592235 10.1093/nar/28.1.235 PMC102472

[7] Rose P.W. , Prlić A., Bi C., Bluhm W.F., Christie C.H., Dutta S., Green R.K., Goodsell D.S., Westbrook J.D., Woo J. et al. The RCSB Protein Data Bank: views of structural biology for basic and applied research and education. Nucleic Acids Res. 2015; 43:D345–D356. 25428375 10.1093/nar/gku1214 PMC4383988

[8] Young J.Y. , Westbrook J.D., Feng Z., Peisach E., Persikova I., Sala R., Sen S., Berrisford J.M., Swaminathan G.J., Oldfield T.J. et al. Worldwide Protein Data Bank biocuration supporting open access to high-quality 3D structural biology data. Database. 2018; 2019:bay002. 10.1093/database/bay002 PMC5804564 29688351

[9] Burley S.K. , Bhikadiya C., Bi C., Bittrich S., Chao H., Chen L., Craig P.A., Crichlow G.V., Dalenberg K., Duarte J.M. et al. RCSB Protein Data Bank (RCSB.org): delivery of experimentally-determined PDB structures alongside one million computed structure models of proteins from artificial intelligence/machine learning. Nucleic Acids Res. 2023; 51:D488–D508. 36420884 10.1093/nar/gkac1077 PMC9825554

[10] Apweiler R. , Bairoch A., Wu C.H., Barker W.C., Boeckmann B., Ferro S., Gasteiger E., Huang H., Lopez R., Magrane M. et al. UniProt: the Universal Protein knowledgebase. Nucleic Acids Res. 2004; 32:D115–D119. 14681372 10.1093/nar/gkh131 PMC308865

[11] The UniProt Consortium Update on activities at the Universal Protein Resource (UniProt) in 2013. Nucleic Acids Res. 2012; 41:D43–D47. 23161681 10.1093/nar/gks1068 PMC3531094

[12] The UniProt Consortium UniProt: a hub for protein information. Nucleic Acids Res. 2015; 43:D204–D212. 25348405 10.1093/nar/gku989 PMC4384041

[13] Gaulton A. , Bellis L.J., Bento A.P., Chambers J., Davies M., Hersey A., Light Y., McGlinchey S., Michalovich D., Al-Lazikani B. et al. ChEMBL: a large-scale bioactivity database for drug discovery. Nucleic Acids Res. 2012; 40:D1100–D1107. 21948594 10.1093/nar/gkr777 PMC3245175

[14] Gaulton A. , Hersey A., Nowotka M., Bento A.P., Chambers J., Mendez D., Mutowo P., Atkinson F., Bellis L.J., Cibrián-Uhalte E. et al. The ChEMBL database in 2017. Nucleic Acids Res. 2017; 45:D945–D954. 27899562 10.1093/nar/gkw1074 PMC5210557

[15] Zdrazil B. , Felix E., Hunter F., Manners E.J., Blackshaw J., Corbett S., de Veij M., Ioannidis H., Lopez D.M., Mosquera J.F. et al. The ChEMBL Database in 2023: a drug discovery platform spanning multiple bioactivity data types and time periods. Nucleic Acids Res. 2024; 52:D1180–D1192. 37933841 10.1093/nar/gkad1004 PMC10767899

[16] Bryant S. PubChem: an information resource linking chemistry and biology. Abst. Pap. Am. Chem. Soc. 2006; 231:80–COMP.

[17] Wang Y. , Xiao J., Suzek T.O., Zhang J., Wang J., Zhou Z., Han L., Karapetyan K., Dracheva S., Shoemaker B.A. et al. PubChem's BioAssay Database. Nucleic Acids Res. 2012; 40:D400–D412. 22140110 10.1093/nar/gkr1132 PMC3245056

[18] Kim S. , Chen J., Cheng T., Gindulyte A., He J., He S., Li Q., Shoemaker B.A., Thiessen P.A., Yu B. et al. PubChem 2019 update: improved access to chemical data. Nucleic Acids Res. 2019; 47:D1102–D1109. 30371825 10.1093/nar/gky1033 PMC6324075

[19] Kim S. , Chen J., Cheng T., Gindulyte A., He J., He S., Li Q., Shoemaker B.A., Thiessen P.A., Yu B. et al. PubChem in 2021: new data content and improved web interfaces. Nucleic Acids Res. 2021; 49:D1388–D1395. 33151290 10.1093/nar/gkaa971 PMC7778930

[20] Kim S. , Chen J., Cheng T., Gindulyte A., He J., He S., Li Q., Shoemaker B.A., Thiessen P.A., Yu B. et al. PubChem 2023 update. Nucleic Acids Res. 2023; 51:D1373–D1380. 36305812 10.1093/nar/gkac956 PMC9825602

[21] Papadatos G. , Davies M., Dedman N., Chambers J., Gaulton A., Siddle J., Koks R., Irvine S.A., Pettersson J., Goncharoff N. et al. SureChEMBL: a large-scale, chemically annotated patent document database. Nucleic Acids Res. 2016; 44:D1220–D1228. 26582922 10.1093/nar/gkv1253 PMC4702887

[22] Morin L. , Weber V., Meijer G.I., Yu F., Staar P.W.J. PatCID: an open-access dataset of chemical structures in patent documents. Nat. Commun. 2024; 15:6532. 39095357 10.1038/s41467-024-50779-y PMC11297020

[23] Wang R. , Fang X., Lu Y., Wang S. The PDBBind database: collection of binding affinities for protein-ligand complexes with known three-dimensional structures. J. Med. Chem. 2004; 47:2977–2980. 15163179 10.1021/jm030580l

[24] Wang R. , Fang X., Lu Y., Yang C.-Y., Wang W. The PDBBind database: methodologies and updates. J. Med. Chem. 2005; 48:4111–4119. 15943484 10.1021/jm048957q

[25] Liu Z. , Li Y., Han L., Li J., Liu J., Zhao Z., Nie W., Liu Y., Wang R. PDB-wide collection of binding data: current status of the PDBbind database. Bioinformatics. 2015; 31:405–412. 25301850 10.1093/bioinformatics/btu626

[26] Durairaj J. , Adeshina Y., Cao Z., Zhang X., Oleinikovas V., Duignan T., McClure Z., Robin X., Rossi E., Zhou G. et al. PLINDER: the protein-ligand interactions dataset and evaluation resource. 2024; bioRxiv doi:17 July 2024, preprint: not peer reviewed 10.1101/2024.07.17.603955.

[27] The UniProt Consortium UniProt: the Universal Protein Knowledgebase in 2023. Nucleic Acids Res. 2023; 51:D523–D531. 36408920 10.1093/nar/gkac1052 PMC9825514

[28] Tropmann K. , Bresinsky M., Forster L., Mönnich D., Buschauer A., Wittmann H.-J., Hübner H., Gmeiner P., Pockes S., Strasser A. Abolishing Dopamine D2long/D3 Receptor Affinity of Subtype-Selective Carbamoylguanidine-Type Histamine H2 Receptor Agonists. J. Med. Chem. 2021; 64:8684–8709. 34110814 10.1021/acs.jmedchem.1c00692

[29] Fabian M.A. , Biggs W.H., Treiber D.K., Atteridge C.E., Azimioara M.D., Benedetti M.G., Carter T.A., Ciceri P., Edeen P.T., Floyd M. et al. A small molecule–kinase interaction map for clinical kinase inhibitors. Nat. Biotechnol. 2005; 23:329–336. 15711537 10.1038/nbt1068

[30] Morgan R.E. , van Staden C.J., Chen Y., Kalyanaraman N., Kalanzi J., Dunn R.T. II, Afshari C.A., Hamadeh H.K A Multifactorial Approach to Hepatobiliary Transporter Assessment Enables Improved Therapeutic Compound Development. Toxicol. Sci. 2013; 136:216–241. 23956101 10.1093/toxsci/kft176

[31] Warner D.J. , Chen H., Cantin L.-D., Kenna J.G., Stahl S., Walker C.L., Noeske T. Mitigating the Inhibition of Human Bile Salt Export Pump by Drugs: opportunities Provided by Physicochemical Property Modulation, In Silico Modeling, and Structural Modification. Drug Metab. Dispos. 2012; 40:2332–2341. 22961681 10.1124/dmd.112.047068

[32] Boess F.G. , Martin I.L. Molecular biology of 5-HT receptors. Neuropharmacology. 1994; 33:275–317. 7984267 10.1016/0028-3908(94)90059-0

[33] Schotte A. , Janssen P.F.M., Gommeren W., Luyten W.H.M.L., Van Gompel P., Lesage A.S., De Loore K., Leysen J.E. Risperidone compared with new and reference antipsychotic drugs: in vitro and in vivo receptor binding. Psychopharmacology (Berl.). 1996; 124:57–73. 8935801 10.1007/BF02245606

[34] Gangjee A. , Lin X. CoMFA and CoMSIA Analyses of Pneumocystis carinii Dihydrofolate Reductase, Toxoplasma gondii Dihydrofolate Reductase, and Rat Liver Dihydrofolate Reductase. J. Med. Chem. 2005; 48:1448–1469. 15743188 10.1021/jm040153n

[35] Anstine D.M. , Isayev O. Generative Models as an Emerging Paradigm in the Chemical Sciences. J. Am. Chem. Soc. 2023; 145:8736–8750. 37052978 10.1021/jacs.2c13467 PMC10141264

[36] Gao W. , Coley C.W. The Synthesizability of Molecules Proposed by Generative Models. J. Chem. Inf. Model. 2020; 60:5714–5723. 32250616 10.1021/acs.jcim.0c00174

[37] Eckmann P. , Sun K., Zhao B., Feng M., Gilson M., Yu R. LIMO: latent Inceptionism for Targeted Molecule Generation. Proceedings of the 39th International Conference on Machine Learning. 2022; PMLR 5777–5792. PMC9527083 36193121

[38] Tang B. , Ewalt J., Ng H.-L. Saxena A.K. Generative AI Models for Drug Discovery. Biophysical and Computational Tools in Drug Discovery, Topics in Medicinal Chemistry. 2021; Cham Springer International Publishing 221–243.

[39] Luo S. , Guan J., Ma J., Peng J. A 3D Generative Model for Structure-Based Drug Design. Advances In Neural Information Processing Systems. 2021; 34:Curran Associates, Inc 6229–6239.

[40] Loeffler H.H. , He J., Tibo A., Janet J.P., Voronov A., Mervin L.H., Engkvist O. Reinvent 4: modern AI–driven generative molecule design. J. Cheminformatics. 2024; 16:20. 10.1186/s13321-024-00812-5 PMC10882833 38383444

[41] Cheng Y. , Gong Y., Liu Y., Song B., Zou Q. Molecular design in drug discovery: a comprehensive review of deep generative models. Brief. Bioinform. 2021; 22:bbab344. 34415297 10.1093/bib/bbab344

[42] Bian Y. , Xie X.-Q. Generative chemistry: drug discovery with deep learning generative models. J. Mol. Model. 2021; 27:71. 33543405 10.1007/s00894-021-04674-8 PMC10984615

[43] Gupta A. , Müller A.T., Huisman B.J.H., Fuchs J.A., Schneider P., Schneider G. Generative Recurrent Networks for De Novo Drug Design. Mol. Inform. 2018; 37:1700111. 29095571 10.1002/minf.201700111 PMC5836943

[44] Walters W.P. , Murcko M. Assessing the impact of generative AI on medicinal chemistry. Nat. Biotechnol. 2020; 38:143–145. 32001834 10.1038/s41587-020-0418-2

[45] Vert J.-P. How will generative AI disrupt data science in drug discovery?. Nat. Biotechnol. 2023; 41:750–751. 37156917 10.1038/s41587-023-01789-6

[46] Bickerton G.R. , Paolini G.V., Besnard J., Muresan S., Hopkins A.L. Quantifying the chemical beauty of drugs. Nat. Chem. 2012; 4:90–98. 22270643 10.1038/nchem.1243 PMC3524573

[47] Ertl P. , Schuffenhauer A. Estimation of synthetic accessibility score of drug-like molecules based on molecular complexity and fragment contributions. J. Cheminform. 2009; 1:8. 20298526 10.1186/1758-2946-1-8 PMC3225829

[48] Weininger D. SMILES, a chemical language and information-system. 1. Introduction to methodology and encoding rules. J. Chem. Inf. Comp. Sci. 1988; 28:31–36.

[49] Heller S.R. , McNaught A., Pletnev I., Stein S., Tchekhovskoi D. InChI, the IUPAC International Chemical Identifier. J. Cheminform. 2015; 7:23. 26136848 10.1186/s13321-015-0068-4 PMC4486400

[50] Responsive Web Design · An A List Apart Article.

[51] Dunbar J.B. , Smith R.D., Yang C.-Y., Ung P.M.-U., Lexa K.W., Khazanov N.A., Stuckey J.A., Wang S., Carlson H.A. CSAR Benchmark Exercise of 2010: selection of the Protein–Ligand Complexes. J. Chem. Inf. Model. 2011; 51:2036–2046. 21728306 10.1021/ci200082t PMC3180202

[52] Carlson H.A. , Smith R.D., Damm-Ganamet K.L., Stuckey J.A., Ahmed A., Convery M.A., Somers D.O., Kranz M., Elkins P.A., Cui G. et al. CSAR 2014: a Benchmark Exercise Using Unpublished Data from Pharma. J. Chem. Inf. Model. 2016; 56:1063–1077. 27149958 10.1021/acs.jcim.5b00523 PMC5228621

[53] Gathiaka S. , Liu S., Chiu M., Yang H., Stuckey J.A., Kang Y.N., Delproposto J., Kubish G., Dunbar J.B., Carlson H.A. et al. D3R Grand Challenge 2015: evaluation of protein-ligand pose and affinity predictions. J. Comput. Aided Mol. Des. 2016; 30:651–668. 27696240 10.1007/s10822-016-9946-8 PMC5562487

[54] Gaieb Z. , Liu S., Gathiaka S., Chiu M., Yang H., Shao C., Feher V.A., Walters W.P., Kuhn B., Rudolph M.G. et al. D3R Grand Challenge 2: blind prediction of protein-ligand poses, affinity rankings, and relative binding free energies. J. Comput. Aided Mol. Des. 2018; 32:1–20. 29204945 10.1007/s10822-017-0088-4 PMC5767524

[55] Gaieb Z. , Parks C.D., Chiu M., Yang H., Shao C., Walters W.P., Lambert M.H., Nevins N., Bembenek S.D., Ameriks M.K. et al. D3R Grand Challenge 3: blind prediction of protein–ligand poses and affinity rankings. J. Comput. Aided Mol. Des. 2019; 33:1–18. 30632055 10.1007/s10822-018-0180-4 PMC6472484

[56] Parks C.D. , Gaieb Z., Chiu M., Yang H., Shao C., Walters W.P., Jansen J.M., McGaughey G., Lewis R.A., Bembenek S.D. et al. D3R grand challenge 4: blind prediction of protein–ligand poses, affinity rankings, and relative binding free energies. J. Comput. Aided Mol. Des. 2020; 34:99–119. 31974851 10.1007/s10822-020-00289-y PMC7261493

[57] Minor D. , Sutton D., Kozbial A., Westbrook B., Burek M., Smorul M. Chronopolis Digital Preservation Network. Int. J. Digit. Curation. 2010; 5:119–133.

[58] Bemis G.W. , Murcko M.A. The Properties of Known Drugs. 1. Molecular Frameworks. J. Med. Chem. 1996; 39:2887–2893. 8709122 10.1021/jm9602928

[59] L’Hours H. , Kleemola M., Leeuw L.de CoreTrustSeal: from academic collaboration to sustainable services. IASSIST Q. 2019; 43:1–17.

[60] CoreTrustSeal Standards and Certification Board CoreTrustSeal Trustworthy Data Repositories Requirements 2020–2022. 2019; 10.5281/zenodo.3638211.

